# Cases of Tic Douloureux, Cured by the Carbonate of Iron

**Published:** 1825-10

**Authors:** Benjamin Hutchinson

**Affiliations:** Surgeon, &c.


### Art. V.—*Cases of Tic Douloureux, cured by the Carbonate of Iron.* Communicated by Benjamin Hutchinson, Esq. Surgeon, &c.

"However, I always like cases; and I like them because I consider that they impress the mind more forcibly than mere words. It is said that History is a mode of teaching Philosophy by a recital of facts."—*Abernethy's Lectures.*

Mr. Wickenden, an eminent surgeon of Birmingham, has communicated the three following interesting cases:—

Mrs. Carless, ætat. sixty-four, Gough-street, Birmingham, during two years has been gradually affected with severe pain in her face. On the 16th of June, 1823, its severity suddenly increased, and, with a few minutes' intermission, continued four days. From her description of the affected parts, the disease appeared to be confined to the ophthalmic branch of the fifth pair of nerves. Calomel purges, with senna and salts, and leeches to the face, were prescribed; afterwards an emetic, succeeded by large doses of opium, two grains of which were given every hour, for six or eight hours, without affording relief. She then took one drachm of the subcarbonate of iron every six hours, and felt perfectly relieved after the sixth dose; and has continued completely free from the disease up to this, the 4th of November, 1823.

In further illustration of this very satisfactory case, it may be permitted to remark, that this lady suffered a pain of the most excruciating kind, concentrated in the suborbitar nerve of the right side of the face. This was aggravated upon the slightest movement, and so fearful was she of touching it, that her face had remained unwashed for weeks together. Of course, eating at all times much increased it; and latterly she had eaten with a wooden spoon, a metal one, if touching the jaw, causing such extreme aggravation of the pain.

Mr. Wickenden is a practitioner whose observation is too correct to render a remark of his of no value. He observes, that formerly he used to give doses of five grains of the iron, but that he never found any effect until he employed the larger quantities now generally adopted.

In the second case drawn up by Mr. Wickenden, the pain was seated in the upper lip, accompanied with frequent convulsive twitches, and was completely cured by drachm doses of the carbonate of iron. The pain in this patient frequently returned, and was as frequently relieved by the ferri subcarbon.

The third case was remarked by most severe pain in one side of the head, particularly behind the ear, and recurring very frequently in the day. After correcting the digestive organs, without affording any relief, the subcarbonate of iron, in drachm doses, perfectly removed the disease.

*Southwell; August.*

